# Characterization of the Mechanical Properties of FFF Structures and Materials: A Review on the Experimental, Computational and Theoretical Approaches

**DOI:** 10.3390/ma12060895

**Published:** 2019-03-18

**Authors:** Enrique Cuan-Urquizo, Eduardo Barocio, Viridiana Tejada-Ortigoza, R. Byron Pipes, Ciro A. Rodriguez, Armando Roman-Flores

**Affiliations:** 1 Tecnologico de Monterrey, Escuela de Ingeniería y Ciencias, Epigmenio González 500 Fracc. San Pablo, Querétaro 76130, Mexico; viri.tejada@tec.mx; 2 School of Materials Engineering, Purdue University, 701 West Stadium Avenue, West Lafayette, IN 47907-2045, USA; ebarocio@purdue.edu (E.B.); bpipes@purdue.edu (R.B.P.); 3 Tecnologico de Monterrey, Escuela de Ingeniería y Ciencias, Av. Eugenio Garza Sada 2501, Monterrey 64849, Mexico; ciro.rodriguez@tec.mx; 4 Laboratorio Nacional de Manufactura Aditiva y Digital (MADIT), Autopista al Aeropuerto, Km., 9.5, Calle Alianza Norte #100, Parque PIIT, Apodaca 66629, Mexico

**Keywords:** additive manufacturing, Fused Deposition Modeling, Fused Filament Fabrication, mechanical characterization

## Abstract

The increase in accessibility of fused filament fabrication (FFF) machines has inspired the scientific community to work towards the understanding of the structural performance of components fabricated with this technology. Numerous attempts to characterize and to estimate the mechanical properties of structures fabricated with FFF have been reported in the literature. Experimental characterization of printed components has been reported extensively. However, few attempts have been made to predict properties of printed structures with computational models, and a lot less work with analytical approximations. As a result, a thorough review of reported experimental characterization and predictive models is presented with the aim of summarizing applicability and limitations of those approaches. Finally, recommendations on practices for characterizing printed materials are given and areas that deserve further research are proposed.

## 1. Introduction

Among all the additive manufacturing (AM) technologies available, the most popular is Fused Deposition Modeling (FDM), also referred to as Fused Filament Fabrication (FFF). This is due to its economic accessibility, ease of use, and variety of materials commercially available [1]. Materials used in FFF are primarily polymers, e.g., polylactic acid (PLA) and acrylonitrile butadiene styrene (ABS), polycarbonate (PC) [2], polyether ether ketone (PEEK) [3,4], ULTEM 9085 [5] and low melting temperature metal alloys [6]. Additionally, other works have upgraded the FFF process to use composites materials [7,8] by reinforcing polymers with carbon fiber [9,10], and for bimodal manufacturing combining FFF and electrospinning [11]. This type of technology was originally used to fabricate prototypes, hence the term rapid-prototyping. However, the current trend is to use AM not only to fabricate prototypes but also to produce end-use components. As a result, understanding the structural behavior of printed components under different loading conditions is important to accelerate the adoption of this manufacturing process.

FFF parts can be used in a wide range of applications, namely Unmanned Aerial Vehicle (UAV) [12], dentistry [13], electrochemical batteries [14], lattice and cellular materials [15,16,17], sandwich structures [18], tissue engineering scaffolds [11,19,20], and even in parts for 3D printers [21]. Even the food industry has explored the fused deposition process for food, for instance, to print with pasta, pork, pizza dough, chocolate, etc. [22,23]. With the increase in number of applications for FFF parts, the understanding of the mechanical properties, namely elastic moduli and strength, has become a priority for these. This has stimulated fundamental research in mechanics and materials inspired by a host of technological applications that demand parts that either need to meet certain mechanical performance or that consist of complex geometries not possible to achieve with conventional subtractive manufacturing methods.

FFF machines are significantly less expensive than other AM machines. An example is the RepRap Project [24], where it is possible to build a functional 3D printer from about £400 (approximately USD 540) [25]. RepRap machines are built with parts fabricated by other printers or by itself, thereby making them economically accessible [26]. On the contrary, Selective Laser Sintering (SLS) or Stereolithography (SLA) can cost 500% more than a FFF machine. Further, the complexity of the fabrication process and the cost of the materials required make the other AM technologies more expensive. While printing, FFF machines can produce fume that can be hazardous, however other AM technologies can be potentially more dangerous due to handling of powders and toxic resins. Currently, multiple brands of FFF machines are straightforward to operate and to transport so that they can be operated from home [27]. However, there is a segment of industrial FFF machines that require larger space and can only be found in research laboratories or industry. Despite the accessibility to FFF machines, limitations such as the quality of the surface finish in printed parts [28,29] and the anisotropy in all the properties of the printed components still impede a wider adoption of this technology [30].

**Figure 1:**
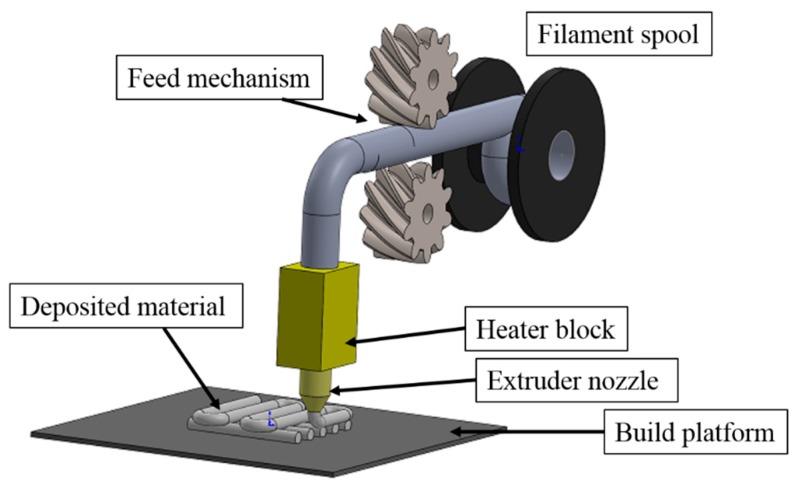
Schematic of the fused filament fabrication technology.

**Figure 2:**
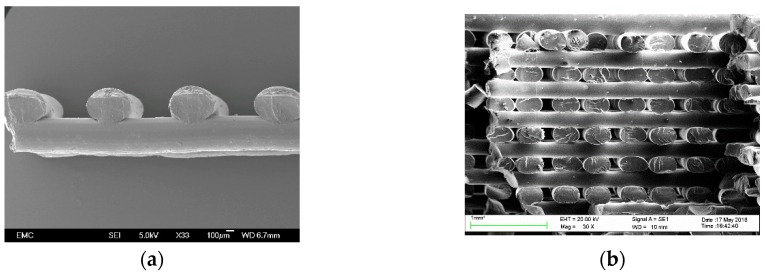
SEM images of FFF parts showing: (**a**) the cross-sectional area of extruded filaments in a two layers array; and (**b**) stacking of several layers.

Fused deposition machines comprise a heater block, an extruder (nozzle) and a platform. The heater and the nozzle are mounted on a moving frame that either moves relative to the build platform or vice versa. The filament feedstock material is pushed into the heater block and partially melted. The material is extruded through the nozzle in the form of a semi-cylindrical filament which is deposited on the platform. This procedure is depicted in Figure 1. The instructions provided by the G-code are processed by the microcontroller that activate the servo-motors to control the location of the nozzle to form the first 2D profile. G-codes consist of lines of instruction containing the coordinates to be followed by the nozzle. Once a layer is extruded, the distance between the nozzle and the platform is increased for the next layer of material to be extruded. This layer-by-layer manufacturing process is carried out until the 3D part is completed. This procedure produces parts composed of the stack of extruded filaments, a close look to the microstructure resulting from this process is shown in the SEM micrograph in Figure 2. Note the extruded filaments once in complete solid state show a semi-elliptic cross-section, and the bonding between layers.

The review of the related literature shows that the study of the mechanical properties of components fabricated by FFF has been restricted primarily to experimental characterization. It has been observed that structural parameters such as filament separation, filament rasters and printing orientation have a greater influence on the mechanical properties of FFF components than manufacturing parameters such as extruder temperature, build platform temperature and printing speed [31,32]. This highlights the importance of understanding the structure–property relation in parts fabricated using this AM-technology. 

The following review is focused on the structure–property relationship and the mechanical characterization of FFF structures and materials. New approaches in manufacturing, such as new materials or combination of materials [33,34], curved layer manufacturing [35,36,37,38] are beyond the scope of this review. This review is structured as follows: experimental characterization including tensile, compression, bending, torsion, and dynamic loading, among others is detailed in Section 2; analytical and semi-analytical approaches are presented in Section 3; computational approaches are included in Section 4; and concluding remarks are given in Section 5.

## 2. Experimental Characterization of Mechanical Properties 

**Figure 3:**
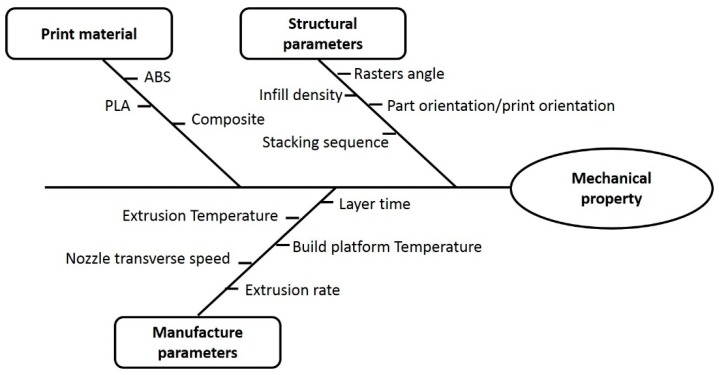
Ishikawa diagram showing the main parameters that have a role on the resulting mechanical properties of FFF.

**Figure 4:**
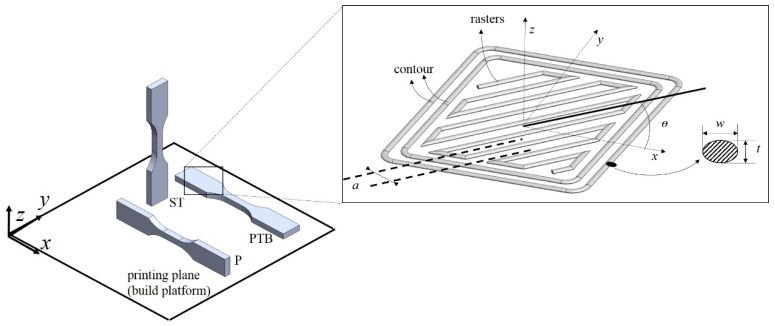
Main structural parameters studied in the mechanical characterization, including the print orientations, raster angle, number of contours, and rasters cross-section.

The FFF process involves various parameters that can be classified into two main groups: (i) manufacturing; and (ii) structural parameters. The first group includes the extrusion temperature, the printing speed or deposition rate, the temperature of the build platform, ambient temperature, etc. The second group includes the gap between printed filaments rasters and layers, the orientation of the rasters and the orientation of the printed part, among others. All these can be modified, and their influence on the resulting mechanical properties must be studied. Those parameters with potential influence on the mechanical properties of FFF are summarized in the Ishikawa diagram in Figure 3. To review characterized mechanical properties in a consistent manner, Figure 4 shows the notation used to refer to the orientation of printed structures and individual unidirectional layers or rasters, respectively. The print orientation where the principal axis is parallel to the stacking direction is denoted as ST. Similarly, PTB refers to specimens printed on the XY plane or directly on the build platform and P refers to specimens printed with the thickness direction laying on the build platform.

Due to the nature of the technology, parts fabricated with this technology will invariably result to be anisotropic. Properties in the plane of deposition (PTB) are significantly different from those along the stacking direction (ST). Additionally, depending on the raster pattern, the mechanical properties at the layer level PTB in Figure 2 may be assumed orthotropic. Furthermore, some of the work reviewed in this paper considered printed structures as an orthotropic material. Characterization using tensile and compression tests, bending and torsion, dynamic loading, mechanical fracture, failure under fatigue and impact is reviewed in this section.

### 2.1. Tensile and Compressive Response

**Figure 5:**
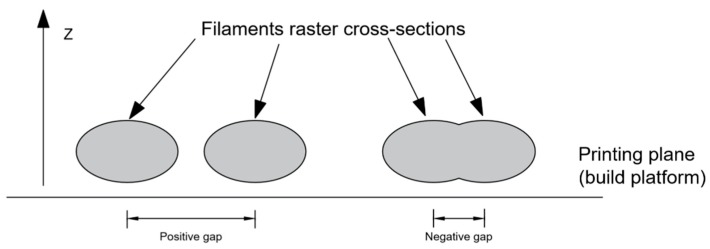
A schematic representation of filament cross-sectional areas, showing the difference between positive and negative gaps.

Among all the experimental studies that deal with the mechanical characterization, tension and compression tests are the most frequently encountered. Some of the earliest works that reported the properties under these loading conditions were by Ahn et al. and Rodriguez et al. [30,39]. The influence of both manufacturing parameters such as extrusion temperature and structural parameters such as raster angle and air gaps on the strength and the elastic properties of printed ABS specimens was reported by Ahn et al. and Rodriguez et al., respectively. Ahn et al. studied quasi-solid samples printed with three different raster angles, θ = 0°, 45° and 90°, and with air gaps varying from a = 0 to negative values a < 0 as represented in Figure 5. Rodriguez et al. [39] employed both negative and positive air gaps for an aligned and skewed stacking of layers. Skewed stacking sequences are those in which adjacent layers have rasters that are staggered and lie at the midpoint between rasters in the same layer. When the samples are quasi-solid, the stiffness suffers almost no variation from the print material properties, as indicated in the mechanical characterization reported by Durgun and Ertan [40]. This is because a quasi-solid FFF sample tends to be a void-free sample as the porosity tends to 0. When samples are fabricated with positive air gaps, reductions of 11–37% in the stiffness have been observed [39]. The case of tensile strength is completely different since this property strongly depends on the defects on the sample, the bonding between layers and the building orientation, as reported in [40]. Zaldivar et al. [41] reported strengths that were 46–85% of the print material strength from 3D ULTEM^®^ dogbones tested under tensile loading. The orientation with the lowest strength was ST or the z-direction according to Figure 4. The raster was not reported by the authors, but the tensile stiffness showed variations within 30% suggesting that the samples were fabricated with 100% density [41]. Between manufacturing and structural parameters, the latter showed a stronger influence on the mechanical properties of the print orientation characterized in this work. This was then further studied by Wittbrodt and Pearce [42]. In [42], the effect of the PLA color and printing temperature on the strength of FFF samples was studied via tensile tests. Samples were fabricated with two different printing temperatures, 190 °C and 215 °C, and with four different colors: white, grey, blue and black. All samples were fabricated with rasters sequences of 0/90° and 100% raster at PTB orientation. While the strength showed no statistical dependence (variation of about 10%) on the PLA color, strength dependency on the crystallinity was more evident. Such a variation in crystallinity is introduced by the variations in cooling history of the printed material.

The influence of layer thickness t (0.2, 0.3, and 0.4 all in mm) on the strength and stiffness for samples with a stacking sequence of 0/90° and 45/−45° was reported by Tymrak et al. [25] for both ABS and PLA. As samples were quasi-solid, values reported for stiffness of ABS and PLA have standard deviations of 72 MPa and 100 MPa, respectively. The strength showed standard deviations for ABS and PLA samples of less than an order of magnitude, 1 MPa and 5 MPa, respectively. The effect of layer thickness t was also studied by Uddin et al. [43]. In this work, layer thickness values of t = 0.09, 0.19, and 0.39 mm were studied on ABS 100% infilled samples. The samples with the thinnest layers showed the highest values for stiffness and strength. When compared with the layer thickness, the infill showed higher influence [44] because porous structures have inferior mechanical properties when compared to their quasi-solid (100% infill) counterparts, as recently concluded in [45].

Influence of the part and print orientation on the strength has also been observed in various works. Several of these reported inferior properties in strength of up to 40% less than those of the print material properties [30,40]. Print orientation resulted to affect more the strength than the stiffness. For example, the tensile strength in the z-direction of a part fabricated in the ST orientation depends on the bonding between layers. On the other hand, when the mechanical properties are tested in the XY plane, the resistance depends on the in-plane properties of each layer and the orientation of each layer in the stack. Cantrell et al. [46] tested a range of different building orientations and reported that, for 100% filled ABS samples, the building orientation shows negligible effect on the stiffness, as expected. 

The combination of design of experiments (DOE) and mathematical-computational methods have also been employed. Sood et al. [47] and Onwubolu and Rayegani [48] considered the influence of the following parameters on the tensile strength: layer thickness t, print orientation, raster angle θ, raster width w, and air gap a. They found that reducing parameters such as w, a, and t results in an increase in the strength properties [48]. A so-called optimal compressive stress of FFF-ABS was obtained as 17.4751 MPa with the values of t = 0.254 mm, print orientation 0.036°, θ = 59.44°, w = 0.442 mm and a = 0.00026 mm [47]. DOE has been used extensively to identify combination of processing and structural parameters that yields the highest mechanical properties. For instance, Deng et al. [49] found that the optimal tensile properties of fused deposited PEEK specimens were observed at a printing speed of 60 mm/s, t = 0.2 mm, temperature of 370 °C and infill density of 40%.

The number of contour rasters, which is indicated in Figure 4, is a parameter that increases the strength and stiffness of the samples [44,50] when the filaments that comprise the contour are aligned in the loading direction. The influence of the contours on a FFF part is directly dependent on the shape of the part. For instance, when using printed dogbone samples, the narrow section is already too thin to have enough distribution of rasters, thereby increasing the contribution of the contours to the mechanical response of the sample. This complicates relating the mechanical properties to the structure, as not enough rasters that define the structure are present in the sample. Hence, the testing standard is another so-called controversial parameter in the mechanical characterization of FFF samples. Further, the mechanical response characterized from printed dogbone with raster patterns oriented in the y-direction is frequently misinterpreted due to the contribution of contours printed along the x-direction. In another investigation, Laureto and Pearce [51] addressed the difference in the strength of tensile PLA specimens utilizing as a reference the ASTM D638 type I and IV. The results show that using Type IV samples may overestimate the ultimate strength when compared with results of Type I. Torrado and Roberson [52] looked at a wider range of ASTM D638 standard types. Type V showed an elongation-to-break value of 0.6–0.9% lower than the rest. The authors attributed this decrement due to variability in the raster. Another important aspect to consider when dealing with geometrical issues is the dimensional error to evaluate the manufacturing accuracy [53].

Rodriguez et al. [39] studied three different stacking sequences in the FFF parts made of ABS. The arrangements tested include: one in which the filament rasters are all oriented in the same direction and with negative gap a; and two skewed stacking sequences, one with negative gap and one with positive (Figure 5). Rodriguez et al. [39] carried out tensile experiments of filament feedstock material to determine the stiffness and strength of the print material and to compare those values with the ones characterized with FFF-ABS samples printed at PTB orientation. From all the arrangements tested, the one with rasters aligned in the loading direction and with negative gaps resulted with the highest stiffness and strength values. The mechanical properties of the FFF parts were found to be inferior to those of the print material. Reductions of 11–37% in the stiffness and 22–37% in the strength were observed and reported by Rodriguez et al. [39]. The largest differences correspond to parts fabricated with positive gaps between filaments. The reduction is attributed to the presence of voids. Hossain et al. [54,55] also reduced the presence of gaps by modifying the default printing parameters, and proposed modifications in the light of improving the ultimate strength in tension. The modifications were increasing the raster and contour width w and reducing the air gap a between rasters. These modifications led to increases in the ultimate strength in the range of 10–30% with respect to the default conditions. The reduction in mechanical properties in FFF when compared with the print material properties is sometimes considered as a drawback. 

Voids within an FFF part can be controlled by changing the infill density as well as by changing their architecture [56]. Further, parts fabricated with voids or positive air gaps are lighter and produced more quickly than fully dense parts, yet with the penalty on mechanical properties, as demonstrated by the multiple works reviewed.

To conclude this section on tensile and compressive properties, a summary of the mechanical properties reported in the literature reviewed above is given in Table 1. Further, the structural and processing conditions listed in Table 1 are limited to the parameters in Figure 3 that are more relevant, and the orientations are consistent with Figure 4. Other approaches for enhancing the mechanical properties of printed components include reinforcing polymers with fillers such as montmorillonite [57], with discontinuous fibers [9,10] and with continuous fibers [58,59,60,61]. Additionally, some researchers have used post-processing techniques to enhance surface quality, strength and tightness [62,63]. Nevertheless, the characterization of parts printed with reinforced polymers and post-processing are beyond the scope of this review paper.

### 2.2. Bending and Torsion Response

Slender structures in flexure are frequently encountered in various applications, such as beams. Despite this, few works are focused on characterizing the flexural properties of FFF beams. Sood et al. [64] reported the strength resulted from three-point bending tests and studied its dependency on parameters such as t, print orientation, θ, w and a. The maximum flexural strength was obtained for print orientation and θ = 0°, t = 0.25 mm, a = 0, and w = 0.5 mm. Wu et al. [65] showed that, using PEEK and having θ = 0°, the bending strength resulted 15% higher than the ABS counterpart. 

Durgun and Ertan [40] reported the flexural strength from three-point bending tests on 100% dense ABS samples fabricated at various angles and orientations. The authors also concluded that surface roughness also plays a significant impact on the flexural strength. Lužanin et al. [66] analyzed the dependence of infill density in the range of 10–30%, layer thickness t in the range of 0.1–0.3 mm and the raster angle θ in the range of 0–60° on the flexural strength of PLA samples. The highest strength reported was for the sample with filaments oriented at 0° and infill of 30%. A raster angle of 0° means that rasters are parallel to the principal axis of the beams, when printed at the PTB orientation. For any value of low infill density, the response is more sensitive to filament orientation in the outermost layers. For θ = 0°, the rasters that run axially withstand the most loading [67]. Somireddy et al. [68] studied the flexural stiffness of ABS samples with the following stacking sequences: 0°/90°, 15°/−75°, 30°/−60°, and 45°/−45. Among the measured data, the maximum variation in stiffness was in the order of 163 MPa among all the different raster angles. These results show a behavior expected for fully dense samples, i.e., convergence to the print material stiffness. The main feature of Somireddy et al.’s work is the prediction based on classic laminate theory [68], therefore it is further reviewed in Section 3. Wagari Gebisa and Lemu [69] characterized the flexural strength and stiffness of FFF samples fabricated using ULTEM 9085 at PTB orientation (see Figure 4). They used a full factorial DOE considering a, w, θ, contour number, and contour width as variables. The two with the most influence on the flexural properties were the raster width and angle. The so-called optimal process parameters obtained in [69] were: a = 0.0 mm, w = 0.7814 mm, θ = 0°, and five contours. These parameters yielded printed structures with a flexural strength of 127 MPa, a flexural modulus of 2400 MPa, and ultimate flexural strain of 0.081.

Chacon et al. [70] ignored the effects of shear in the transverse deflection, and reported the flexural modulus based on classic beam theory for PLA samples with a = 0. Printing speed was the parameter that showed the most influential role in the flexural stiffness. Nevertheless, as the air gap used approached zero, the flexural stiffness resulted with maximum variation of 33% among all the combinations of print orientation, layer thickness and feed rate. The effect of air gaps greater than zero was analyzed by Cuan-Urquizo and Bhaskar [67]. They studied the structure–property relationship for the 0°/90° stacking sequence of raster angles of PLA samples, but to several raster densities. The approach in [67] is mainly theoretical, hence it is reviewed in Section 3. Although it is beyond the scope of this paper, it is worth mentioning that recently Kuznetsov et al. [71] reported the ultimate fracture strength for tubular (0% infill) samples under three-point bending.

The understanding of the torsional properties of FFF parts is an open issue that has been almost not addressed in the available literature. Few works have been reported along these lines, for instance Balderrama-Armendariz et al. [72] studied 100% dense ABS samples fabricated at different print orientations and with rasters at different angles, unidirectional and 45°/−45°, under twist loadings. They characterized the shear modulus, ultimate strength, and fracture strain. In the following order, fracture strain, strength and stiffness are more sensitive to print orientation. Balderrama-Armendariz et al. [72], when comparing FFF-ABS samples with injection molding, found all variables measured resulted in similar values, except for fracture strain. This is why FFF samples are less ductile than injection molding samples. 

### 2.3. Dynamic Loading Response

Despite the numerous works of characterizing properties through static experiments, the dynamic properties have been less popular. Applications such as UAVs can be structurally subjected to dynamic loading scenarios. Therefore, the works reviewed in this section gain relevance especially for end-use components fabricated through FFF.

Domingo-Espin et al. [73] characterized the influence of nozzle diameter, number of contours, and air gap on the dynamic response of PC samples. The relationship among extruder diameter, layer thickness, contour and raster width, number of contours, and air gap, on the storage and loss moduli were characterized using Dynamic Mechanical Analysis (DMA). The influence of amplitude, frequency and temperature were also studied. The results of loss modulus reported by Domingo-Espin et al. show that a decrease in raster not only increases the compliance of the structure but also the damping characteristics. Amplitude, frequency, and temperature resulted to affect the damping capacity. Among all the parameters, the number of contours was the most influential since they add stiffness to the part. This was also observed by Mohamed et al. for printed specimens tested using the DMA [74,75,76]. The dynamic stiffness of PC-ABS samples was studied using DMA. The so-called optimal values for the parameters were obtained via a graphical optimization and these were close to the lower bounds for thickness, and air gap.

A frequency sweep from 10 Hz to 100 Hz using three different temperatures was performed by Arivazhagan and Masood [77] on ABS samples fabricated with fully dense top and bottom layers, and with different raster patterns. The raster patterns tested were unidirectional and bi-directional, quasi-solid and partially filled. Storage and loss moduli resulted inferior for partially filled rasters than for quasi-solid ones. Loss and storage moduli have a strong dependence on temperature and thus a steep decrease in these two properties was characterized as temperature increased. 

Jami et al. [78] compared the quasi-static stress–strain response of FFF-ABS samples under compression, with the dynamic response using a Split Hopkinson pressure bar. The dynamic response showed higher dependency on print orientation, while orientation showed almost no effect on quasi-static loading conditions. 

### 2.4. Fracture Properties

An important aspect in characterizing components and materials is not only knowing when failure occurs but also to understand the failure mechanisms. Linear elastic fracture mechanics methods developed for homogeneous solids might not be applicable to structures produced by FFF. These structures cannot be treated as homogenous solids, even in 100% infilled samples voids and material anisotropy are present. Torrado Perez et al. [79] studied the fracture surface of pure ABS and ABS matrix composites in 100% infilled samples. The addition of reinforcing elements resulted in FFF samples with higher properties than the pure ABS. The reinforcements reported, namely jute fiber, thermo plastic elastomer and TiO_2_, also changed the ductile behavior of ABS to brittle.

Aliheidari et al. [80] fabricated 100% infilled double cantilever beam samples to study mode-I fracture of ABS samples. Samples with uniaxial filaments were fabricated so that the filaments were aligned to the longitudinal axis of the cantilever beams. They compared the strength of the FFF samples fabricated at three different nozzle temperatures: 210, 230, and 240 °C. They employed a J-integral method to estimate the mode-I energy release rate for fracture between layers and for fracture inside layers. Further, resistance plots were reported for both the interlayer and intra-layer fracture. The results showed that the higher is the temperature, the higher is the resistance to fracture. The authors state this indicated an enhanced bonding between layers and favorable meso-structure features such as layer thickness and raster pattern. Again, these experimental results highlight the strong dependence that properties in the stacking direction have on processing conditions.

Hart and Wetzel [81] studied the effect of raster angle orientation on the fracture properties of 100% infilled ABS samples. Horizontal (PTB) and vertical (ST) samples were printed to study the fracture across and between layers, respectively. Results demonstrated that the critical elastic–plastic strain energy release rate required to propagate a crack across layers of printed material FFF was approximately an order of magnitude greater than the energy required to propagate cracks between FFF layers of printed specimens. Brittle behavior was observed during fracture between layers while ductile response was observed for fracture across the layers indicating that the elastic–plastic response of the material depends on the orientation of the rasters with respect to the crack-tip. Similar results but on PLA samples fabricated with uniaxial raster angles (0° and 90°) and biaxial raster angles (0/90°) were reported by Arbeiter et al. [82]. 

### 2.5. Fatigue, Failure under Cyclic Loading

The repetitive loading on structural elements results in failure due to fatigue. As many of FFF parts are intended to be used as machine components, they are likely to be subjected to periodic loading with high frequency. Some works on the failure under cyclic loading were found and are summarized here.

Gomez-Gras et al. [83] studied two raster patterns, square and hexagonal lattice, under cyclic loading using a beam rotating machine (GUNT WP 140). The influence of t, infill density, nozzle diameter, and printing velocity, on the fatigue response of PLA samples fabricated was studied. From all the data obtained from the testing, they presented the S-N curve for the optimal parameters obtained. These optimal parameters were: 75% of raster, hexagonal raster pattern, nozzle diameter of 0.5 mm, and t = 0.3 mm. Note that in [83], samples with 100% raster were not studied, therefore the optimal value for raster resulted to be the highest value tested 75%. Puigoriol-Frocada et al. [84] built S-N curves from experimental results on PC samples fabricated at the print orientations shown in Figure 4. Among the three print orientations, PTB and P exhibited longest cyclic fatigue life, whereas ST print orientation failed rapidly under cyclic loading. On the one hand, the load in the print orientations PTB and P is carried by the raster structure and the contours. On the other hand, in samples fabricated in the ST print orientation, the load is carried by the bond between layers.

The tensile fatigue life of FFF samples was also investigated by Ziemian et al. [85] by testing uniaxial and bidirectional samples made of ABS with zero air gap a. With regards to the observations made on the fatigue tests, bi-directional samples showed fatigue behavior similar to reinforced composites. Further, the principal fatigue failure mechanisms observed were filament cracking, delamination, and changes in the geometry of the voids. 

### 2.6. Structural Response under Impact

ABS shows different fracture behavior when the samples are tested at different temperatures [86], especially at temperatures above the Tg of the polymer, therefore the study of FFF-ABS gains relevance. One of the most common experiments to characterize the impact resistance is the Izod impact testing. To carry out this test, a pivoting arm is raised to a specific height to provide constant potential energy. Subsequently to releasing arm, the arm impacts the specimen and the energy absorbed by the specimen is estimated from the height the arm swings back. One of the earliest works that used the Izod testing for FFF samples was done by Es-Said [87]. In this work, ABS uniaxial samples with raster angles of 0° and 90° and bidirectional with a stacking sequence of 45°/−45° were fabricated with a = 0. In the uniaxial samples with rasters angle at 0°, the extruded filaments are oriented parallel to the principal axis of the samples. The length in the uniaxial 90° orientation samples is defined by the stack of layers. The highest value of absorbed energy was reported for the 0° uniaxial raster angle, while the lowest was observed for the 90°. Similar results were reported by Roberson et al. [88].

Wang et al. [89] also studied the influence of different build platform temperatures on the impact strength by utilizing the Izod standard. In this work, PLA samples were fabricated at PTB print orientation and 100% infilled but at different bed temperatures and layer thicknesses. Results indicate that reducing layer thickness to a minimum value of 0.2 mm and increasing the printing temperature to a maximum of 160 °C produces printed samples with impact strength that exceeds the one measured from injected molded PLA samples. Further, a higher bed temperature results in more crystallinity developed in the PLA, which results in higher strength under impact loading. On the contrary, samples manufactured at lower bed temperatures (30 °C) show impact strengths around 20% lower than injected PLA samples. This suggests that crystallinity plays an important role in the impact strength of printed structures.

A key aspect in impact testing is the stress sensibility to the notch on the samples. Therefore, Roberson et al. [88] studied this effect by fabricating notches utilizing two methods: first, by printing the notch during the specimen fabrication and, second, by machining the notch in a previously printed sample. The results of both methods show no statistical difference, concluding the correct use of the standards for impact characterization. 

Another work that focused on the mechanical response under impact loading is found in [90]. Tsouknidas et al. [90] studied the shock absorption properties using a drop tower system. Cylindrical PLA samples were fabricated with 25% and 50% infill densities at ST print orientation. Their results show that solid samples outperform in terms of structural integrity the partially filled ones. Nevertheless, the ratio of absorbed energy over the volume fraction is higher for porous samples. 

## 3. Modeling with Theoretical Methods: Analytical and Semi-Analytical

This section reviews analytical and empirical methods as well as their combination that have been developed to estimate the mechanical response of printed structures.

### 3.1. Approaches Based on Laminate Plate Theory

**Figure 6:**
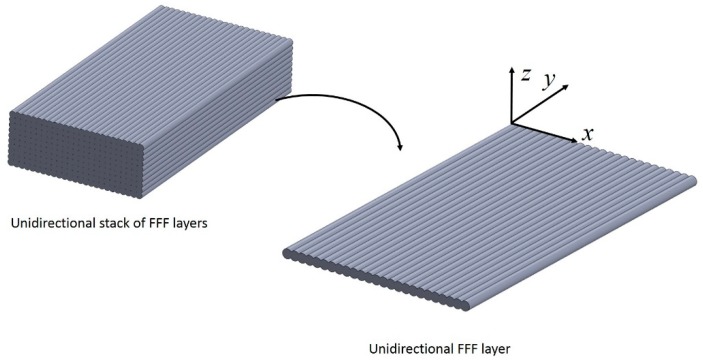
Schematic representation of a unidirectional stack of FFF layers.

The layers that form the FFF part can be treated as laminae, as depicted in Figure 6. This suggests that Classic Laminate Theory (CLT) may be employed to estimate the mechanical properties of FFF parts in restricted scenarios. Classic laminate theory has been combined with experimental tests to study the mechanical properties of FFF components. Kulkarni and Tutta [91], Li et al. [92], and, more recently, Casavola et al. [93] and Ziemian et al. [85] used similar approaches to obtain elastic constants for a printed lamina through experimental characterization. Kulkarni and Dutta [91] were perhaps the first to use CLT to predict the mechanical properties of parts fabricated with FFF. The stiffness matrix was populated with experimentally measured elastic properties. The properties were obtained from tensile test on unidirectional samples. Different raster angles were tested including longitudinal, transverse and 45°. Shear modulus was obtained from the tensile test on the samples with rasters oriented at 45°. Li et al. [92] followed a similar procedure and presented SEM images of the cross-section of samples fabricated with various values of air gaps. These micrographs were used to calculate the void density of the printed samples based on an empirical model. Casavola et al. [93], obtained the elastic moduli from tensile tests of unidirectional quasi-solid samples. They obtained the longitudinal Young’s modulus and Poisson’s ratio from samples where the rasters are aligned to the loading direction. The researchers obtained the transverse Young’s modulus of filaments running parallel and bonded together by testing them in a direction perpendicular to that of the rasters. They measured the shear modulus using off-axis tests of specimens oriented at 45° from the loading direction. The laminate model was used to predict the Young’s modulus of a sample fabricated with the following stacking sequence: 30°/−30°/0°/−30°/30°. The predictions made were in good agreement with the experimentally measured modulus, specifically with a maximum difference of 6.6% in the worst cases. Similarly, Ziemian et al. [85] used CLT constructed from properties measured from unidirectional samples to predict the stiffness of a printed structure with multiple layer orientations. Additionally, the tensile fatigue life of FFF samples was studied by the same authors. For the fatigue tests, bi-directional samples showed fatigue behavior, similar to that of reinforced composites. The main fatigue failure mechanisms reported were filament cracking, delamination, and changes in the geometry of the voids.

Classic laminate approaches are restricted to parts fabricated with negative or zero separation between filaments. Kulkarni and Tutta [91] and Li et al. [92] both accounted for the effect of the inevitable voids on the stiffness of the printed parts by incorporating stochastic factors in their formulations. The approach of Casavola et al. [93] and Zeimian et al. [85] was limited to parts fabricated with 100% infill; no separation between the extruded filaments was specified. To obtain the complete stiffness matrix, the Young’s moduli of a single layer in the longitudinal and transverse directions of the filaments rasters are needed. The stiffness in the transverse direction is only obtained when the filaments within the layer interact, in other words, when all the filaments are connected in the transverse direction. The prediction of the mechanical properties using CLT fails when filaments are extruded with an air gap between each other as the stiffness in the transverse direction becomes negligible [94]. For example, if a single FFF layer, such as the one shown in Figure 6, is loaded along the y-axis, expressions to predict the stiffness are readily obtained. However, the stiffness along the x-axis depends on the in-plane bonding of filaments. Consider now the supposed case where filaments in the FFF layer have a positive air gap; when loaded along the y-axis, they offer no resistance to the load. 

### 3.2. Micromechanics Approach

**Figure 7:**
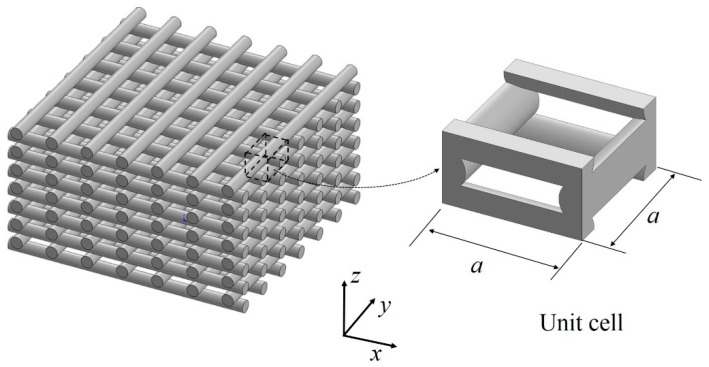
Schematic representation of the FFF raster lattice structure. Rasters are represented with cylinders, and the unit cell of such lattice is shown as an extraction from the lattice.

To address the shortcoming of CLT for predicting mechanical response of structures printed with partial infill, micromechanics-based approaches have been developed. This approach focuses on analyzing repeating unit cells rather than layers and assumes that filament rasters are perfectly bonded within a unit cell [93,94]. Figure 7 shows an example of a unit cell analyzed in a micromechanics approach. Croccolo et al. [95] proposed an analytical model to predict the mechanical response of 45° off-axis specimens printed with zero air gap between rasters. The analytical model was based on considering the inclined filaments as inclined truss members. The adhesive force between rasters was determined empirically. The model predicted stiffness and strength with errors of about 4%.

Huang and Singamneni [96] studied the sensitivity of the effective stiffness and strength on unidirectional samples fabricated with various raster angles. Huang and Singamneni proposed an analytical model based on the plane stress response of perfectly bonded layers. In addition, the coalescence between rasters was considered by them in another work [97]. Because rasters are bonded within the layer, Huang et al. considered the filament cross-sections to be square with elliptical fillets in their analysis. The properties studied, stiffness, strength and Poisson’s ratio, resulted to be highly sensitive to the coalescence. For various levels of coalescence, differences from 80% to 100% were observed in the stiffness and Poisson’s ratio for the most extreme cases analyzed by the authors.

The presence of voids (a > 0) in FFF parts results in a reduction of weight and manufacturing time but complicates the prediction of the mechanical properties. Two analytical models reviewed in this section [95,96] were developed for fully-filled FFF structures. One of the few works that have related the effective properties of the printed part to the structure formed in the presence of voids in a FFF part was done by Cuan-Urquizo et al. [67,98]. Cuan-Urquizo considered the FFF raster structure as a cellular material [99] and treated FFF parts as being composed of lattice materials. In [98], the properties along the raster direction were studied by means of analytical prediction and finite element simulations. The analytical model was derived on the assumption that, when the lattice shown in Figure 7 is loaded along one of the principal axes, only the rasters parallel to the loading direction contribute to carrying the load. The analytical model presented in [98] is based on the rule of mixtures. This was compared with finite element predictions, showing errors less than 10%. Details of the FE model are reviewed in Section 4.

In [67], the flexural properties of FFF beams were studied for a structure of rasters with angles and sequence of 0/90°. The primary deformation mechanisms identified for individual filaments were stretching and bending. In the case of pure bending, raster filaments that run parallel to the principal axis respond in tension or compression depending on their relative position with respect to the neutral axis. The bending model proposed was derived based on the later observation. Then, when incorporating a shear inclusive model, the deformation mechanism of the filament when the lattice structure undergoes shear stresses was observed as flexure. Filaments bend at an effective length is given by the distance between rasters of adjacent layers (air gap). 

This fundamental micromechanics observations of the filaments enables the derivation of analytical expressions for the structure–property relationship. The effective Young’s modulus and the effective shear modulus resulted in linear and cubic relationships with the relative infill density, respectively. Details of the derivations and the expressions can be found in [67,98]. Models presented in [67,98] are compared with finite element simulations and reviewed in Section 4. 

#### Micromechanics of Tissue Engineering Woodpile Scaffolds

The structured form in the raster of FFF parts consists of the stack of extruded filaments, as shown in Figure 7. This structure, known as woodpile structure, is also encountered in Tissue Engineering (TE), where scaffolds are needed to withstand the ingrowth of tissue, and at the same time serve as a porous matrix for cell proliferation. The fact that these scaffolds need to be structurally efficient has inspired the research community to study their mechanical properties [100,101,102]. It should be noted that these works are not reported with the aim of understanding the mechanical properties of FFF components, but, if TE scaffolds have the same architecture as FFF components, these approaches show relevant results in light of understanding the structure–property relationship of extruded based AM technologies, and therefore are included here.

Norato and Wagoner-Johnson [101] studied the woodpile material arrangement using the unit cell approach and presented analytical models to predict the effective Young’s modulus along the z-direction. Norato and Wagoner-Johnson modeled the raster filaments as cylinders. The model consists of the interpolation between the plane stress and the plane strain responses of a disc and a cylinder under diametrically opposed loads. The constants for the interpolation-based model were obtained from simulations using a geometry projection numerical model. The apparent shear modulus of woodpile materials was studied analytically by Roberge and Norato [103] and Cuan-Urquizo and Bhaskar [67]. Further, the deformation mechanism has been identified to be filaments flexure when the whole structure is subject to pure shear. As mentioned, this results in a cubic relation with the relative density [67,103].

The models for the effective properties presented in [101,103] show a dependence on the fraction of the area shared across adjacent filaments, and these predict the stiffness with an error of less than 6%. This area fraction is known as overlap, and is shown in [67] to be a highly influential parameter on the transverse deflection of FFF beams due to shear deformations. More specifically, the stiffness of the cell will respond to the kinematic constraint assumed in the overlaps. On the one hand, if all degrees of freedom are assumed to be coupled at the overlap between two connected filaments, then the stiffest response is obtained. On the other hand, if rotation is allowed between two connected filaments, then the overall response of the structure becomes more compliant than the former case.

## 4. Modeling with Computational Methods

Traditionally, computational simulations and finite element models have been used to estimate the structural performance of structures. Due to the complexity of the material arrangement naturally obtained in FFF, this appears to be a good option for their analysis. Computational simulations to predict the mechanical properties of FFF have been carried out using finite element analysis (FEA). Finite element models found in the literature for printed structures can be classified into two main groups. First, printed parts are modeled as solid parts with some homogenized effective properties. This means that the rasters are not modeled explicitly [104]. The second group includes those works where the microstructure has been modeled explicitly with as much resemblance of the structure obtained in FFF as possible [105,106]. These groups are reviewed below.

The use of computation and finite element method is a powerful tool to predict the mechanical properties, but also to model the 3D printing process itself. FEM has been used to model the FFF manufacturing process [107,108,109] as well as the large-scale variation of this process [110,111,112,113,114]. Nevertheless, process simulation of the FFF process is beyond the scope of this review paper. This manuscript is rather focused on reviewing approaches that utilize FEM to predict the mechanical response of printed structures, while disregarding stresses induced in the manufacturing process.

### 4.1. Finite Element Homogenized Models

The ability to estimate effective macro-scale material properties requires that a heterogeneous material can be represented with a volume element or unit cell that shows periodicity across a body. A two-step homogenization approach was utilized by Liu and Shapiro for analyzing structures that are much larger than the printed road width, w and layer thickness, t [115]. In such situations, the estimated effective material properties may be used for subsequent mechanical simulations of global elastic response, vibrations and modal analysis.

Domingo-Espin et al. [104] carried out experimental characterization via tensile tests of the orthotropic elastic constants of FFF printed samples. These samples were fabricated along different print orientations, fully dense, and the raster angle sequences of 45°/−45°. They utilized the characterized elastic constants in FEA models to predict the mechanical response of structural elements. In [104], a structural part with “L” geometry was subjected to static loading. Such geometry allows exposing the part to both bending and torsion. The difference between FEA predictions and experimental data was 7%. Experimental results were also compared to predictions made with a FEA model informed with isotropic properties obtained by averaging the orthotropic properties. The predictions made with this approach resulted in variations of 7% with respect to the experimental data. These results show again that the degree of anisotropy in the elastic properties is less in parts fabricated with 100% infill density.

The use of effective properties in homogenizing FEA models appears to be a good option for understanding and predicting the structural performance of FFF parts with complex macro geometries. This tool for predicting structural performance of FFF parts appears to be a reliable option, providing good characterization of the properties to feed the FEA model are obtained. However, this methodology is limited in terms of capturing the wide range of possibilities for printing orientations and raster patterns that FFF offers. Further, homogenized models obscure important aspects of the micromechanics such as the deformation mechanisms and stress localization, which are fundamental for predicting local failure mechanisms. 

### 4.2. Finite Element Microstructure Models

The works reviewed in this section use explicit structures formed by extruded filaments. To model these, different types of elements have been used to mesh the structures. Cuan-Urquizo et al. [98] used 1D finite beam elements to model the rasters. Since adjacent layers of rasters are not running in the same plane, beam elements with infinite stiffness were used to represent this offset. In [93], the FE model is tested only under tensile loading. To explore the advantages of this type of FE mesh, simulations of more complex loadings are not addressed in that work. This type of approaches requires less computational resources as opposed to 3D meshing models. However, they hide important aspects of micromechanics, such as stress concentration effects and the influence of bonding between layers. The latter is incorporated in [67]. Cuan-Urquizo and Bhaskar modeled the bonding between layers as a finite overlap between two orthogonally adhering cylinders which represents the filament rasters. In other words, overlap between adjacent cylinders was represented by having a patch of volume shared between them. They showed [67] that, as the overlap increases, the bonding behaves as a welded-joint rather than a pin joint, thereby adding stiffness to the overall response. FE models in [67] are meshed using tetrahedral FE elements. The prediction of the flexural response results with less than 10% error.

Section 2.1 shows that the geometry and dimensions of the samples used to characterize the mechanical properties can influence the properties measured [51,52]. This was further studied using FEM by Wendt et al. [105]. Wendt et al. used FEM to estimate the zone where the samples for tensile testing (ISO 3167) are likely to fail. The CAD models built to be used in FEM consist of circular cross-sections being swept along the rasters. This type of geometry modeling allows the FEM models to be more representative of the printed structure as opposed to homogenized FE models reviewed in the previous section. These results are in good agreement to the previously reviewed works included in Section 2.1.

CAD representations of rasters as swept constant cross-sectional areas were also employed by Somireddy and Czekanski [106]. They used uniaxial FE models similar to those shown in Figure 6 to obtain the elastic constants. These models represented the cross-section of the rasters as ellipses and included overlaps between adjacent layers and rasters. The elastic constant obtained were the Poisson’s ratio (νxy), the shear modulus (Gxy) and the elastic moduli along each of axes in the XY plane or printing plane (Ex and Ey). The FE models were subjected to unit displacement upon applying a tensile load in the respect direction. The total strain energy *U* obtained from the FEA model was utilized to estimate the elastic constant *E_ii_* using the expression:

*U*~(1/2)*E_ii_ԑ_ii_^2^V*, (1)

where *V* is the volume undergoing deformation, and *ε_ii_* is the strain in the ii direction. For the shear modulus, the FE model was built with the rasters oriented at 45°. Thus, upon the application of a tensile load, the model response is in shear. The elastic constants were then used to inform an analytical model based on CLT.

In a different work, Somireddy et al. [116] followed a similar procedure to estimate the elastic constants of the orthotropic FFF material. In [116], as opposed to the work presented in [106], the authors built FE models of a unit cell or Representative Volume Element (RVE). The geometry of the RVE consisted of ellipsoidal cross-section areas with overlaps to represent the bonding between rasters and layers. Two RVEs were modeled representing parts in the PTB and ST orientations, respectively. These RVEs were subjected to tensile loading, and the homogenized properties obtained from the RVE analysis were used to model more complicated structures. While predicting the response of structures printed in the PTB configuration could be approached with CLT, vertical structures such as the ST configuration require specific application of the results from their corresponding RVE.

Even though the works presented in [105,106,116] are complex enough to capture aspects of micromechanics of FFF materials, their application is restricted to FFF components printed with 100% infill density as their models only account for rasters bonded in and out the plane. The study of the mechanics of porous structures built by extrusion techniques using FEM can be found also for TE scaffolds, which are reviewed in the following subsection.

Since inter-layer properties are strongly dependent on processing conditions, Barocio et al. [112,113] developed a method to predict the evolution of inter-layer properties during the printing process, thereby closing the gap between processing conditions and part performance. This approach has been validated for fiber reinforced semi-crystalline polymers and consists of coupling the temperature evolution, polymer crystallization and melting, and polymer diffusion.

Utilizing FE approaches, the thermomechanical response of a printed mold designed for operating at temperatures up to 180 °C was analyzed by the group of Brenken and Barocio [117,118]. This approach has been demonstrated to be effective for printed composite materials and captures explicitly the anisotropy in printed parts by assigning orthotropic material properties to each individual raster.

#### Finite Element Simulations of Tissue Engineering Woodpile Scaffolds

As mentioned above, the fact that TE scaffolds involve structures similar to the ones produced naturally in FFF makes the attempts to understand their mechanical properties relevant to this review. Among the attempts to predict the mechanical properties, Miranda et al. [119,120] made use of FE by modeling the woodpile structure as a stack of cylinders with a fixed overlap to represent the bonding. The cylinders were then meshed using tetrahedral FE elements. Miranda et al. [119,120] studied the mechanical properties of calcium scaffolds for tissue engineering applications using FEA and laboratory experiments. In [119], they subjected the scaffolds to compressive tests to study their strength. Two different ceramic materials were compared (β-tricalcium phosphate and hydroxyapatite). The failure of these scaffolds was attributed to the micro-cracks in the ceramic filaments. In [120], they carried out a stress analysis using the stresses obtained from a FEA model to estimate the strength properties of a scaffold structure. The FEA models consisted of a stack of struts modeled as cylinders with a constant overlap between them. The models were subjected to compressive, tensile and shear loading. The stress contours were studied for each loading, showing a directional dependence. The shear simulations showed high concentration of stress at the joints, indicating a high potential for failure at these locations. The scaffolds tested here were of high relative density, having the filaments at a distance slightly bigger than their diameter. This ratio of filament separation to filament diameter complicates the development of analytical micromechanics models, thus the analysis carried out by the authors was restricted to FEA predictions [120].

In addition, the compressive response and its relation with the intra-layer bonding was investigated by Naghieh et al. [102] using a finite element model of PLA bone scaffolds. They fabricated the scaffolds using FFF and showed that the effective Young’s modulus is strongly dependent to this parameter: stiffness increases as the overlap increases. 

## 5. Conclusions

A review on the different attempts to study the mechanical properties of FFF is addressed here. Numerous works including experimental, theoretical and computational approaches for estimating the mechanical response of printed structures under different loading conditions are reviewed. Further, general conclusions in terms of applicability, advantages and disadvantages of the approaches reviewed are summarized in Table 2. Finally, general concluding remarks and recommendations for future work are presented below.

- The stiffness of structures printed with amorphous polymers such as ABS converge to those of the print material for quasi-solid samples, i.e., those fabricated with 100% raster.
- Works that tend to cover a wide number of parameters use DOE to study their influence on the mechanical properties. However, the specific contribution of each parameter tends to be hidden due to the number of variables and unknowns intrinsic to the process.
- Works done via experimental testing have exposed the need for testing standards for AM characterization. The sensitivity to structural parameters of the measured data makes the geometry of the samples a crucial aspect to consider. Comparisons of testing samples, for example for impact or tensile testing, could hide or increase the influence of certain parameters, e.g., contour rasters.
- Extensive experimental characterization of FFF structures has been carried out, however the properties measured with this approach cannot be readily used to model real printed structures. Instead, the approach that is recommended is the one characterizing the properties of the representative volume along with boundary conditions that best describes the heterogeneity of the printed part. For instance, in an extruded-base scaffold structure, this would correspond to the unit cell of the lattice repeating across the structure, whereas, in the case of a printed part combining solid layers and partial infill, characterizing the properties by sections could be a good compromise between capturing each filament explicitly and homogenizing the structure as a whole.
- Most of the works found deal with quasi-solid samples. Nevertheless, one of the advantages of AM technologies is that the weight reduction that can be obtained by fabricating partially filled parts. A gap in the knowledge is found in the characterization of the mechanical properties of FFF parts fabricated with low densities at various types of loadings.
- The applicability of CLT approaches is mainly restricted to quasi-solid samples since these rely on the assumption that properties are continuous inside each layer. Unit cell approaches showed to be more accurate for partially-filled structures, which are characterized by discontinuities in the properties for each layer.
- For static mechanical properties, the samples with the lowest porosity resulted in the highest mechanical properties. However, the dynamic properties showed an improvement in the damping behavior caused by increasing the porosity in the printed structure.
- The review of the use of FEM in the analysis of the mechanical properties showed that, when modeling the microstructure, more details of the deformation mechanisms are captured. However, more computational resources are demanded, as the number of FE element increases.
- Properties such as inter-layer bonding, which are sensitive to processing conditions, are required to model failure of the printed structures. Hence, process simulations are suggested to close the gap between manufacturing process and prediction of mechanical performance of printed parts. This allows a better understanding of the FFF process and it could lead to a more accurate prediction of the resulting properties.

Various approaches have been used to characterize the mechanical properties of FFF parts and structures. Individual conclusions of the most relevant works have been summarized in this review paper. Almost no approach can be used individually, rather a combination of characterization results should be considered to have reliable and safe estimation of the properties. While pure experimental works may hide the individual influence of each manufacture or structural parameter, analytical approaches always rely on assumptions. Nevertheless, experimental works can give a quicker estimation of mechanical performance. Moreover, analytical approaches contribute directly to the structure–property relationship, which could be used to tailor mechanical properties. Computational approaches could be the best options available provided there is a consistent procedure to obtain the properties to feed the models.

The freedom for design and manufacturing enabled through FFF has also created a demand for predictive tools, not only capable of predicting the performance of printed components, but also capable of anticipating deformation, stresses and failure resulting from the manufacturing process. This type of general solutions is emerging, as reviewed in this work, yet still deserves more research.

## Figures and Tables

**Table 1:** Summary of the works for the tensile characterization.

	*Structural Parameters*							*Manufacturing Parameters*				
Authors	Material	Infill (%)	Airgap (mm)	Part Orientation	Raster Angle (deg)	Layer Thickness (mm)	Raster Width (mm)	Temp. (°C)	Trans. Speed (mm/s)	Test	Mech. Properties	Main Conclusions
Ahn et al. [30]	ABS	100	0, −0.002	PTB, ST	0, 0/90, 45/−45, 90	-	-	260,280	-	Tension, compression	Strength	Reductions in the range of 20–88%
Rodriguez et al. [39]	ABS	100	−0.0254	PTB	0,90	-	-	270	12.7	Tension	Stiffness	Reductions in the range of 11–37%
Zaldivar et al. [41]	ULTEM 9085	-	-	PTB, ST, 45°	-	-	-	-	-	Tension	Strength	Reductions in the range of 15–54%
Wittbrodt and Pearce [42]	PLA	100	-	PTB	0/90	-	-	190	-	Tension	Strength, strain	Minor variation in strength, more significance in crystallinity
Tymrak et al. [25]	ABS/PLA	100	-	PTB	0/90, 45/−45	0.4, 0.3, 0.2	-	-	-	Tension	Strength, stiffness	Standard deviation in strength for ABS and PLA of 0.15 and 0.85 MPa, and in stiffness of 34 and 41 MPa
Uddin et al. [43]	ABS	100	-	PTB, ST, P	-	0.09, 0.19, 0.39	-	245	-	Tension, compression	Strength, stiffness, strain	Lowest values for layer thickness showed higher stiffness and strength
Cantrell et al. [46]	ABS/PC	100	-	PTB, ST, P	45/−45, 30/−60, 15/−75, 0/90	0.254	-	-	-	Tensions, shear	Strength, stiffness	Varying raster orientation results in anisotropic properties in the printing plane
Sood et al. [47]	ABS	-	0, 0.004, 0.008	PTB	0, 30, 60	0.127, 0.178, 0.254	0.4064	-	-	Compression	Strength	Optimal compressive stress was 17.4751 MPa with the values of layer thickness 0.254 mm, orientation 0.036 deg, raster angle 59.44 deg, raster width 0.442 mm and air gap 0.00026 mm
Onwubolu and Rayegani [48]	ABS	-	−0.56134	PTB, ST	0, 45	0.127–0.3302	0.2032–0.5588	-	-	Tension	Strength	Optimal parameters: Layer thickness 0.778 mm, raster angle 45°, width 0.5588 mm, airgap 0.0025 mm
Deng et al. [49]	PEEK	20,40,60	-	-	-	0.2, 0.25, 0.30	-	350, 360,370	20, 40, 60	Tension	Strength, stiffness, strain	Optimal properties were obtained at 60 mm/s, layer thickness 0.2 mm, temperature 370 °C
Laureto and Pearce [51]	PLA	100	-	PTB, ST	-	-	-	175–230	30–200	Tension	Strength	Geometry of the samples have an impact on the measured properties.
Torrado and Roberson [52]	ABS	100	-	PTB, ST	0, 0/90	0.1, 0.2, 0.3	-	230	-	Tension	Strength, strain	The need for testing standards for FFF is exposed
Hossain et al. [54,55]	PC	-	0, −0.103	PTB, ST, P	0/90, 30/−60, 45/−45	-	0.4, 0.8	-	-	Tension	Strength, stiffness, strain	Strength was increased in all orientations, 16% in PTB 7% in P, and 22% in ST

**Table 2:** Comparison of the different characterization methods.

Characterization Method	Applicability	Advantages	Disadvantages
*Experimental* [30,31,32,33,34,35,36,37,38,39,40,41,42,43,44,45,46,47,48,49,50,51,52,53,54,55,56,57,58,59,60,61,62,63,64,65,66,67,68,69,70,71,72,73,74,75,76,77,78,79,80,81,82,83,84,85,86,87,88,89,90]	Fully-filled and partially filled-structures	- Inclusion of wide range of variables/parameters - Can be used to study physics that theory neglects.	- Compromises understanding of structure–property relationship - Requires numerous tests - Restricted to conventional loading scenarios
*CLT* [91,92,93,94]	Fully-filled structures	- Different stacking sequences - Saves in number of tests	- Mainly restricted to fully-dense structures - Requires characterization of materials constants
*Micromechanics* [95,96,97,98]	Fully-filled and partially filled-structures	- Structure–property relationship - Id. of deformation mechanisms	- Uses theoretical assumptions - Restricted to simple macro geometries
*Unit cell* [67,101]	Partially-filled structures and lattice rastered structures	- Structure–property relationship - Id. of deformation mechanisms	- Uses theoretical assumptions - Restricted to simple macro geometries
*FEA* [67,98,104,105,106,107,116]	Fully-filled and partially filled-structures	- Modeling of complex geometries - Saves in number of tests - Predicts without the need of manufacturing - Can be employed to simulate the process	- Compromises understanding of structure–property relationship - Can hide deformation mechanisms

## Abbreviations

ABS	Acrylonitrile Butadiene Styrene
AM	Additive Manufacturing
CAD	Computer-Aided Design
CLT	Classic Laminate Theory
DMA	Dynamic Mechanical Analysis
DOE	Design of experiments
FDM	Fused Deposition Modeling
FE	Finite element
FEA	Finite Element Analysis
FEM	Finite Element Model
FFF	Fused Filament Fabrication
P	Specimens printed with the thickness direction laying on the build platform
PC	Polycarbonate
PEEK	Polyether Ether Ketone
PLA	Polylactic acid
PTB	Printed on the XY plane
RVE	Representative Volume Element
SEM	Scanning electron microscope
ST	Printed parallel to the stacking direction
TE	Tissue Engineering

## References

[1] Tanikella N.G., Wittbrodt B., Pearce J.M. (2017). Tensile strength of commercial polymer materials for fused filament fabrication 3D printing. Addit. Manuf..

[2] Hill N., Haghi M. (2014). Deposition direction-dependent failure criteria for fused deposition modeling polycarbonate. Rapid Prototyp. J..

[3] Chia H.N., Wu B.M. (2015). Recent advances in 3D printing of biomaterials. J. Biol. Eng..

[4] Arif M.F., Kumar S., Varadarajan K.M., Cantwell W.J. (2018). Performance of biocompatible PEEK processed by fused deposition additive manufacturing. Mater. Des..

[5] Bagsik A., Schoppner V. (2011). Mechanical properties of fused deposition modeling parts manufactured with ULTEM * 9085. ANTEC..

[6] Mireles J., Espalin D., Roberson D., Zinniel B., Medina F., Wicker R. Fused Deposition Modeling of Metals. Proceedings of the Solid Freeform Fabrication Symposium.

[7] Brenken B., Barocio E., Favaloro A., Kunc V., Pipes R.B. (2018). Fused filament fabrication of fiber-reinforced polymers: A review. Addit. Manuf..

[8] Ning F., Cong W., Qiu J., Wei J., Wang S. (2015). Additive manufacturing of carbon fiber reinforced thermoplastic composites using fused deposition modeling. Compos. Part B Eng..

[9] Tekinalp H.L., Kunc V., Velez-Garcia G.M., Duty C.E., Love L.J., Naskar A.K., Blue C.A., Ozcan S. (2014). Highly oriented carbon fiber—Polymer composites via additive manufacturing. Compos. Sci. Technol..

[10] Love L.J., Elliott A.M., Post B.K., Smith R.J., Blue C.A. (2014). The importance of carbon fiber to polymer additive manufacturing. J. Mater. Res..

[11] Lara-Padilla H., Mendoza-Buenrostro C., Cardenas D., Rodriguez-Garcia A., Rodriguez C.A. (2017). Influence of controlled cooling in bimodal scaffold fabrication using polymers with different melting temperatures. Materials.

[12] Brischetto S., Ciano A., Ferro C.G. (2016). A multipurpose modular drone with adjustable arms produced via the FDM additive manufacturing process. Curved Layer. Struct..

[13] Chen H., Yang X., Chen L., Wang Y., Sun Y. (2016). Application of FDM three-dimensional printing technology in the digital manufacture of custom edentulous mandible trays. Sci. Rep..

[14] Arenas L.F., Walsh F.C., de León C.P. (2015). 3D-Printing of Redox Flow Batteries for Energy Storage: A Rapid Prototype Laboratory Cell. ECS J. Solid State Sci. Technol..

[15] Ravari M.K., Kadkhodaei M., Badrossamay M., Rezaei R. (2014). Numerical investigation on mechanical properties of cellular lattice structures fabricated by fused deposition modeling. Int. J. Mech. Sci..

[16] Tabacu S., Ducu C. (2018). Experimental testing and numerical analysis of FDM multi-cell inserts and hybrid structures. Thin-Walled Struct..

[17] Baikerikar P.J., Turner C.J. Comparison of As-Built FEA Simulations and Experimental Results for Additively Manufactured Dogbone Geometries. Proceedings of the 37th Computers and Information in Engineering Conference.

[18] Brischetto S., Ferro C.G., Torre R., Maggiore P. (2018). 3D FDM production and mechanical behavior of polymeric sandwich specimens embedding classical and honeycomb cores. Curved Layer. Struct..

[19] Hutmacher D.W., Schantz T., Zein I., Ng K.W., Teoh S.H., Tan K.C. (2001). Mechanical properties and cell cultural response of polycaprolactone scaffolds designed and fabricated via fused deposition modeling. J. Biomed. Mater. Res..

[20] Chin Ang K., Fai Leong K., Kai Chua C., Chandrasekaran M. (2006). Investigation of the mechanical properties and porosity relationships in fused deposition modelling-fabricated porous structures. Rapid Prototyp. J..

[21] Jones R., Haufe P., Sells E., Iravani P., Olliver V., Palmer C., Bowyer A. (2011). Reprap—The replicating rapid prototype. Robotica.

[22] Godoi F.C., Prakash S., Bhandari B.R. (2016). 3d printing technologies applied for food design: Status and prospects. J. Food Eng..

[23] Sun J., Zhou W., Huang D., Fuh J.Y., Hong G.S. (2015). An Overview of 3D Printing Technologies for Food Fabrication. Food Bioprocess Technol..

[24] Bowyer A. (2014). 3D printing and humanity’s first imperfect replicator. 3D Print. Addit. Manuf..

[25] Tymrak B.M., Kreiger M., Pearce J.M. (2014). Mechanical properties of components fabricated with open-source 3-D printers under realistic environmental conditions. Mater. Des..

[26] Bogue R. (2013). 3D printing: The dawn of a new era in manufacturing?. Assem. Autom..

[27] Roberson D.A., Espalin D., Wicker R.B. (2013). 3D printer selection: A decision-making evaluation and ranking model. Virtual Phys. Prototyp..

[28] Agarwala M.K., Jamalabad V.R., Langrana N.A., Safari A., Whalen P.J., Danforth S.C. (1996). Structural quality of parts processed by fused deposition. Rapid Prototyp. J..

[29] Weeren R.V., Agarwala M., Jamalabad V.R., Bandyopadhyay A., Vaidyanathan R., Langrana N., Safari A., Whalen P., Danforth S.C., Ballard C. (1995). Quality of Parts Processed by Fused Deposition. Proc. Solid Free. Fabr. Symp..

[30] Ahn S.H., Montero M., Odell D., Roundy S., Wright P.K. (2002). Wright, Anisotropic material properties of fused deposition modeling ABS. Rapid Prototyp. J..

[31] Montero M., Roundy S., Odell D., Ahn S.H., Wright P.K. Material Characterization of Fused Deposition Modeling (FDM) ABS by Designed Experiments. Proceedings of the Rapid Prototyping & Manufacturing Conference.

[32] Thrimurthulu K.P.P.M., Pandey P.M., Reddy N.V. (2004). Optimum part deposition orientation in fused deposition modeling. Int. J. Mach. Tools Manuf..

[33] Masood S.H., Song W.Q. (2004). Development of new metal/polymer materials for rapid tooling using Fused deposition modelling. Mater. Des..

[34] Carneiro O.S., Silva A.F., Gomes R. (2015). Fused deposition modeling with polypropylene. Mater. Des..

[35] Lee W.C., Wei C.C., Chung S.C. (2014). Chung, Development of a hybrid rapid prototyping system using low-cost fused deposition modeling and five-axis machining. J. Mater. Process. Technol..

[36] Chakraborty D., Reddy B.A., Choudhury A.R. (2008). Extruder path generation for Curved Layer Fused Deposition Modeling. CAD Comput. Aided Des..

[37] Jin Y., Du J., He Y., Fu G. (2017). Modeling and process planning for curved layer fused deposition. Int. J. Adv. Manuf. Technol..

[38] Lim S., Buswell R.A., Valentine P.J., Piker D., Austin S.A., De Kestelier X. (2016). Modelling curved-layered printing paths for fabricating large-scale construction components. Addit. Manuf..

[39] Rodríguez J.F., Thomas J.P., Renaud J.E. (2001). Mechanical behavior of acrylonitrile butadiene styrene (ABS) fused deposition materials. Experimental investigation. Rapid Prototyp. J..

[40] Durgun I., Ertan R. (2014). Experimental investigation of FDM process for improvement of mechanical properties and production cost. Rapid Prototyp. J..

[41] Zaldivar R.J., Witkin D.B., McLouth T., Patel D.N., Schmitt K., Nokes J.P. (2017). Influence of processing and orientation print effects on the mechanical and thermal behavior of 3D-Printed ULTEM^®^9085 Material. Addit. Manuf..

[42] Wittbrodt B., Pearce J.M. (2015). The effects of PLA color on material properties of 3-D printed components. Addit. Manuf..

[43] Uddin M.S., Sidek M.F.R., Faizal M.A., Ghomashchi R., Pramanik A. (2017). Evaluating Mechanical Properties and Failure Mechanisms of Fused Deposition Modeling Acrylonitrile Butadiene Styrene Parts. J. Manuf. Sci. Eng..

[44] Griffiths C.A., Howarth J., Rowbotham G.D.A., Rees A. (2016). Effect of Build Parameters on Processing Efficiency and Material Performance in Fused Deposition Modelling. Procedia CIRP.

[45] Rodríguez-Panes A., Claver J., Camacho A. (2018). The influence of manufacturing parameters on the mechanical behaviour of PLA and ABS pieces manufactured by FDM: A comparative analysis. Materials.

[46] Cantrell J.T., Rohde S., Damiani D., Gurnani R., DiSandro L., Anton J., Young A., Jerez A., Steinbach D., Kroese C. (2017). Experimental Characterization of the Mechanical Properties of 3D-Printed ABS and Polycarbonate Parts. Rapid Prototyp. J..

[47] Sood A.K., Ohdar R.K., Mahapatra S.S. (2012). Experimental investigation and empirical modelling of FDM process for compressive strength improvement. J. Adv. Res..

[48] Onwubolu G.C., Rayegani F. (2014). Characterization and Optimization of Mechanical Properties of ABS Parts Manufactured by the Fused Deposition Modelling Process. Int. J. Man. Eng..

[49] Deng X., Zeng Z., Peng B., Yan S., Ke W. (2018). Mechanical properties optimization of poly-ether-ether-ketone via fused deposition modeling. Materials.

[50] Sukindar N.A.B., Ariffin M.K.A.B.M., Baharudin B.H.T.B., Jaafar C.N.A.B., Ismail M.I.S.B. (2017). Analysis on the impact process parameters on tensile strength using 3d printer repetier-host software. ARPN J. Eng. Appl. Sci..

[51] Laureto J.J., Pearce J.M. (2018). Anisotropic mechanical property variance between ASTM D638-14 type i and type iv fused filament fabricated specimens. Polym. Test..

[52] Torrado A.R., Roberson D.A. (2016). Failure analysis and anisotropy evaluation of 3D-printed tensile test specimens of different geometries and print raster patterns. J. Fail. Anal. Prev..

[53] Messimer S.L., Pereira T.R., Patterson A.E., Lubna M., Drozda F.O. (2019). Full-Density Fused Deposition Modeling Dimensional Error as a Function of Raster Angle and Build Orientation: Large Dataset for Eleven Materials. J. Manuf. Mat. Proc..

[54] Hossain M.S., Ramos J., Espalin D., Perez M., Wicker R. (2013). Improving tensile mechanical properties of FDM-manufactured specimens via modifying build parameters. Int. S. F. F. Symp..

[55] Hossain M.S., Espalin D., Ramos J., Perez M., Wicker R. (2014). Improved mechanical properties of fused deposition modeling-manufactured parts through build parameter modifications. J. Manuf. Sci. Eng..

[56] Fernandez-Vicente M., Calle W., Ferrandiz S., Conejero A. (2016). Effect of infill parameters on tensile mechanical behavior in desktop 3D printing. 3D Print. Addit. Manuf..

[57] Weng Z., Wang J., Senthil T., Wu L. (2016). Mechanical and thermal properties of ABS/montmorillonite nanocomposites for fused deposition modeling 3D printing. Mater. Des..

[58] Mark G.T., Gozdz A.S. (2014). Apparatus for fiber reinforced additive manufacturing. U.S. Patent.

[59] Van Der Klift F., Koga Y., Todoroki A., Ueda M., Hirano Y., Matsuzaki R. (2016). 3D printing of continuous carbon fibre reinforced thermo-plastic (CFRTP) tensile test specimens. Open J. Compos. Mater..

[60] Melenka G.W., Cheung B.K., Schofield J.S., Dawson M.R., Carey J.P. (2016). Evaluation and prediction of the tensile properties of continuous fiber-reinforced 3D printed structures. Compos. Struct..

[61] Li N., Li Y., Liu S. (2016). Rapid prototyping of continuous carbon fiber reinforced polylactic acid composites by 3D printing. J. Mater. Process. Tech..

[62] Jo K.H., Jeong Y.S., Lee J.H., Lee S.H. (2016). A study of post-processing methods for improving the tightness of a part fabricated by fused deposition modeling. Int. J. Prec. Eng. Manuf..

[63] Nguyen T.K., Lee B.K. (2018). Post-processing of FDM parts to improve surface and thermal properties. Rapid Prototyp. J..

[64] Sood A.K., Ohdar R.K., Mahapatra S.S. (2010). Parametric appraisal of mechanical property of fused deposition modelling processed parts. Mater. Des..

[65] Wu W., Geng P., Li G., Zhao D., Zhang H., Zhao J. (2015). Influence of layer thickness and raster angle on the mechanical properties of 3D-printed PEEK and a comparative mechanical study between PEEK and ABS. Materials.

[66] Lužanin O., Movrin D., Plančak M. (2014). Effect of Layer Thickness, Deposition Angle, and Infill on Maximum Flexural Force in Fdm-Built Specimens. J. Technol. Plast..

[67] Cuan-Urquizo E., Bhaskar A. (2018). Flexural elasticity of woodpile lattice beams. Eur. J. Mech. A Solids.

[68] Somireddy M., De Moraes D.A., Czekanski A. Flexural behavior of fdm parts: Experimental, analytical and numerical study. Proceedings of the 28th Annual International Solid Freeform Fabrication Symposium—An Additive Manufacturing Conference.

[69] Gebisa A., Lemu H. (2018). Investigating effects of Fused-Deposition Modeling (FDM) processing parameters on flexural properties of ULTEM 9085 using designed experiment. Materials.

[70] Chacón J.M., Caminero M.A., García-Plaza E., Núñez P.J. (2017). Additive manufacturing of PLA structures using fused deposition modelling: Effect of process parameters on mechanical properties and their optimal selection. Mater. Des..

[71] Kuznetsov V., Solonin A., Urzhumtsev O., Schilling R., Tavitov A. (2018). Strength of PLA components fabricated with fused deposition technology using a desktop 3D printer as a function of geometrical parameters of the process. Polymers.

[72] Balderrama-Armendariz C.O., MacDonald E., Espalin D., Cortes-Saenz D., Wicker R., Maldonado-Macias A. (2018). Torsion analysis of the anisotropic behavior of FDM technology. Int. J. Adv. Manuf. Technol..

[73] Domingo-Espin M., Borros S., Agullo N., Garcia-Granada A.A., Reyes G. (2014). Influence of building parameters on the dynamic mechanical properties of polycarbonate fused deposition modeling parts. 3D Print. Addit. Manuf..

[74] Mohamed O.A., Masood S.H., Bhowmik J.L. (2017). Experimental investigation for dynamic stiffness and dimensional accuracy of FDM manufactured part using IV-Optimal response surface design. Rapid Prototyp. J..

[75] Mohamed O.A., Masood S.H., Bhowmik J.L. (2016). Experimental investigations of process parameters influence on rheological behavior and dynamic mechanical properties of FDM manufactured parts. Mater. Manuf. Process..

[76] Mohamed O.A., Masood S.H., Bhowmik J.L. (2016). Experimental investigation of the influence of fabrication conditions on dynamic viscoelastic properties of PC-ABS processed parts by FDM process. IOP Conf. Ser. Mater. Sci. Eng..

[77] Arivazhagan A., Saleem A., Masood S.H., Nikzad M., Jagadeesh K.A. (2014). Study of dynamic mechanical properties of fused deposition modelling processed. Int. J. Eng. Res. Appl..

[78] Jami H., Masood S.H., Song W.Q. (2013). Dynamic response of FDM made ABS parts in different part orientations. Adv. Mater. Res..

[79] Torrado Perez A.R., Roberson D.A., Wicker R.B. (2014). Fracture surface analysis of 3D-printed tensile specimens of novel ABS-based materials. J. Fail. Anal. Prev..

[80] Aliheidari N., Tripuraneni R., Ameli A., Nadimpalli S. (2017). Fracture resistance measurement of fused deposition modeling 3D printed polymers. Polym. Test..

[81] Hart K.R., Wetzel E.D. (2017). Fracture behavior of additively manufactured acrylonitrile butadiene styrene (ABS) materials. Eng. Fract. Mech..

[82] Arbeiter F., Spoerk M., Wiener J., Gosch A., Pinter G. (2018). Fracture mechanical characterization and lifetime estimation of near-homogeneous components produced by fused filament fabrication. Polym. Test..

[83] Gomez-Gras G., Jerez-Mesa R., Travieso-Rodriguez J.A., Lluma-Fuentes J. (2018). Fatigue performance of fused filament fabrication PLA specimens. Mater. Des..

[84] Puigoriol-Forcada J.M., Alsina A., Salazar-Martín A.G., Gomez-Gras G., Pérez M.A. (2018). Flexural fatigue properties of polycarbonate fused-deposition modelling specimens. Mater. Des..

[85] Ziemian C.W., Ziemian R.D., Haile K.V. (2016). Characterization of stiffness degradation caused by fatigue damage of additive manufactured parts. Mater. Des..

[86] Newmann L.V., Williams J.G. (1978). The impact behavior of ABS over a range of temperatures. Polym. Eng. Sci..

[87] Es-Said O.S., Foyos J., Noorani R., Mendelson M., Marloth R., Pregger B.A. (2000). Effect of layer orientation on mechanical properties of rapid prototyped samples. Mater. Manuf. Process..

[88] Roberson D.A., Perez A.R.T., Shemelya C.M., Rivera A., MacDonald E., Wicker R.B. (2015). Comparison of stress concentrator fabrication for 3D printed polymeric izod impact test specimens. Addit. Manuf..

[89] Wang L., Gramlich W.M., Gardner D.J. (2017). Improving the impact strength of Poly(lactic acid) (PLA) in fused layer modeling (FLM). Polymer.

[90] Tsouknidas A., Pantazopoulos M., Katsoulis I., Fasnakis D., Maropoulos S., Michailidis N. (2016). Impact absorption capacity of 3D-printed components fabricated by fused deposition modelling. Mater. Des..

[91] Kulkarni P., Dutta D. (1999). Deposition Strategies and Resulting Part Stiffnesses in Fused Deposition Modeling. J. Manuf. Sci. Eng..

[92] Li L., Sun Q., Bellehumeur C., Gu P. (2002). composite modeling and analysis for fabrication of fdm prototypes with locally controlled properties. J. Manuf. Process..

[93] Casavola C., Cazzato A., Moramarco V., Pappalettere C. (2016). Orthotropic mechanical properties of fused deposition modelling parts described by classical laminate theory. Mater. Des..

[94] Magalhães L.C., Volpato N., Luersen M.A. (2014). Evaluation of stiffness and strength in fused deposition sandwich specimens. J. Brazilian Soc. Mech. Sci. Eng..

[95] Croccolo D., De Agostinis M., Olmi G. (2013). Experimental characterization and analytical modelling of the mechanical behaviour of fused deposition processed parts made of ABS-M30. Comput. Mater. Sci..

[96] Huang B., Singamneni S. (2014). Raster angle mechanics in fused deposition modelling. J. Compos. Mater..

[97] Huang B., Singamneni S. (2014). Adaptive slicing and speed-and time-dependent consolidation mechanisms in fused deposition modeling. Proc. Inst. Mech. Eng. Part B J. Eng. Manuf..

[98] Cuan-Urquizo E., Yang S., Bhaskar A. (2015). Mechanical characterisation of additively manufactured material having lattice microstructure. IOP Conf. Ser. Mater. Sci. Eng..

[99] Gibson L.J., Ashby M.F., Schajer G.S., Robertson C.I. (1982). The mechanics of two-dimensional cellular materials. Proc. R. Soc. A Math. Phys. Eng. Sci..

[100] Zein I., Hutmacher D.W., Tan K.C., Teoh S.H. (2002). Fused deposition modeling of novel scaffold architectures for tissue engineering applications. Biomaterials.

[101] Norato J.A., Wagoner Johnson A.J. (2011). Computational and cellular solids approach to the stiffness-based design of bone scaffolds. J. Biomech. Eng..

[102] Naghieh S., Karamooz Ravari M.R., Badrossamay M., Foroozmehr E., Kadkhodaei M. (2016). Numerical investigation of the mechanical properties of the additive manufactured bone scaffolds fabricated by FDM: The effect of layer penetration and post-heating. J. Mech. Behav. Biomed. Mater..

[103] Roberge J., Norato J. (2018). Computational design of curvilinear bone scaffolds fabricated via direct ink writing. CAD Comput. Aided Des..

[104] Domingo-Espin M., Puigoriol-Forcada J.M., Garcia-Granada A.-A., Llumà J., Borros S., Reyes G. (2015). Mechanical property characterization and simulation of fused deposition modeling *Polycarbonate parts*. Mater. Des..

[105] Wendt C., Valerga A.P., Droste O., Batista M., Marcos M. (2017). FEM based evaluation of fused layer modelling monolayers in tensile testing. Procedia Manuf..

[106] Somireddy M., Czekanski A. (2017). Mechanical characterization of additively manufactured parts by FE modeling of mesostructured. J. Manuf. Mater. Process..

[107] Zhang Y., Chou Y. (2006). Three-dimensional finite element analysis simulations of the fused deposition modelling process. Proc. Inst. Mech. Eng. Part B J. Eng. Manuf..

[108] Nickel A.H., Barnett D.M., Prinz F.B. (2001). Thermal stresses and deposition patterns in layered manufacturing. Mater. Sci. Eng. A.

[109] Zhang Y., Chou K. (2008). A parametric study of part distortions in fused deposition modelling using three-dimensional finite element analysis. Proc. Inst. Mech. Eng. Part B J. Eng. Manuf..

[110] Favaloro A.J., Brenken B., Barocio E., Pipes R.B. Simulation of polymeric composites additive manufacturing using Abaqus. Proceedings of the Dassault Systemes’ Science in the Age of Experience.

[111] Brenken B., Favaloro A., Barocio E., Pipes R.B. (2017). Simulation of semi-crystalline composite tooling made by extrusion deposition additive manufacturing. Int. SAMPE Tech. Conf..

[112] Barocio E., Brenken B., Favaloro A.J., Ramirez M., Ramirez J., Pipes R.B. Prediction of the degree of bonding in the extrusion deposition additive manufacturing process of semi-crystalline polymer composites. Proceedings of the Dassault Systemes’ Science in the Age of Experience.

[113] Barocio E., Brenken B., Favaloro A.J., Ramirez J., Kunc V., Pipes R.B. Fusion bonding simulations of semi-crystalline polymer composites in the extrusion deposition additive manufacturing process. Proceedings of the American Society for Composites.

[114] Brenken B., Favaloro A., Barocio E., Kunc V., Pipes R.B. Thermoviscoelasticity in extrusion deposition additive manufacturing process simulations. Proceedings of the American Society for Composites.

[115] Liu X., Shapiro V. (2016). Homogenization of material properties in additively manufactured structures. CAD Comput. Aided Des..

[116] Somireddy M., Czekanski A., Singh C.V. (2018). Development of constitutive material model of 3D printed structure via FDM. Mater. Today Commun..

[117] Barocio E., Brenken B., Favaloro A., Pipes R.B. Extrusion deposition additive manufacturing of composite molds for high-temperature applications. Proceedings of the Int. SAMPE Tech. Conf..

[118] Brenken B., Barocio E., Favaloro A.J., Pipes R.B. Simulation of semi-crystalline composites in the extrusion deposition additive manufacturing process. Proceedings of the American Society for Composites.

[119] Miranda P., Pajares A., Saiz E., Tomsia A.P., Guiberteau F. (2007). Fracture modes under uniaxial compression in hydroxyapatite scaffolds fabricated by robocasting. J. Biomed. Mater. Res..

[120] Miranda P., Pajares A., Guiberteau F. (2008). Finite element modeling as a tool for predicting the fracture behavior of robocast scaffolds. Acta Biomater..

